# Volatile hydrocarbons inhibit methanogenic crude oil degradation

**DOI:** 10.3389/fmicb.2014.00131

**Published:** 2014-04-03

**Authors:** Angela Sherry, Russell J. Grant, Carolyn M. Aitken, D. Martin Jones, Ian M. Head, Neil D. Gray

**Affiliations:** School of Civil Engineering and Geosciences, Newcastle UniversityNewcastle upon Tyne, UK

**Keywords:** methanogenic, oil biodegradation, volatile hydrocarbons, non-weathered oil, weathered oil, *n*-alkanes

## Abstract

Methanogenic degradation of crude oil in subsurface sediments occurs slowly, but without the need for exogenous electron acceptors, is sustained for long periods and has enormous economic and environmental consequences. Here we show that volatile hydrocarbons are inhibitory to methanogenic oil biodegradation by comparing degradation of an artificially weathered crude oil with volatile hydrocarbons removed, with the same oil that was not weathered. Volatile hydrocarbons (*n*C_5_–*n*C_10_, methylcyclohexane, benzene, toluene, and xylenes) were quantified in the headspace of microcosms. Aliphatic (*n*-alkanes *n*C_12_–*n*C_34_) and aromatic hydrocarbons (4-methylbiphenyl, 3-methylbiphenyl, 2-methylnaphthalene, 1-methylnaphthalene) were quantified in the total hydrocarbon fraction extracted from the microcosms. 16S rRNA genes from key microorganisms known to play an important role in methanogenic alkane degradation (*Smithella* and *Methanomicrobiales*) were quantified by quantitative PCR. Methane production from degradation of weathered oil in microcosms was rapid (1.1 ± 0.1 μmol CH_4_/g sediment/day) with stoichiometric yields consistent with degradation of heavier *n*-alkanes (*n*C_12_–*n*C_34_). For non-weathered oil, degradation rates in microcosms were significantly lower (0.4 ± 0.3 μmol CH_4_/g sediment/day). This indicated that volatile hydrocarbons present in the non-weathered oil inhibit, but do not completely halt, methanogenic alkane biodegradation. These findings are significant with respect to rates of biodegradation of crude oils with abundant volatile hydrocarbons in anoxic, sulphate-depleted subsurface environments, such as contaminated marine sediments which have been entrained below the sulfate-reduction zone, as well as crude oil biodegradation in petroleum reservoirs and contaminated aquifers.

## Introduction

Hydrocarbons are common in many subsurface environments (Gray et al., 2010) where their degradation to methane by methanogenic microbial consortia is known to occur (Zengler et al., 1999; Anderson and Lovley, 2000; Townsend et al., 2003; Siddique et al., 2006; Gieg et al., 2008, 2010; Jones et al., 2008; Wang et al., 2011). Rates of hydrocarbon degradation are generally lower under anoxic conditions than under oxic conditions (Grishchenkova et al., 2000), and methanogenic hydrocarbon degradation demonstrably supports the lowest rates (Townsend et al., 2003; Jones et al., 2008). For example, during hydrocarbon degradation linked to sulfate reduction lag times are shorter and degradation rates faster in comparison to analogous methanogenic systems (Townsend et al., 2003; Jones et al., 2008; Sherry et al., 2013). This difference is related to the free energy yield of different terminal anaerobic processes. Hexadecane oxidation coupled to sulfate reduction yields −556.9 kJ/mol hexadecane, whereas, methanogenic degradation yields −371.8 kJ/mol. Nevertheless, anaerobic respiratory processes require exogenous electron sinks whereas methanogenesis continues in their absence (Townsend et al., 2003; Gray et al., 2010). Even in hydrocarbon contaminated marine and estuarine sediments initial concentrations of sulfate are likely to be rapidly depleted (Teske, 2010). Accordingly, understanding the controls on methanogenic hydrocarbon degradation is important for assessing the fate of hydrocarbons in anoxic environments. Methanogenic oil degradation is, for instance, most likely responsible for the formation of a large proportion of the world's vast deposits of heavy oil (Head et al., 2003; Townsend et al., 2003; Jones et al., 2008; Gray et al., 2010) and, is an important factor in the natural attenuation of hydrocarbon plumes in contaminated aquifers (Amos et al., 2005; Baedecker et al., 2011). Further, stimulation of methanogenic oil degradation in petroleum reservoirs reaching the end of their normal production lifetime has been proposed as a means to re-pressurize spent reservoirs and recover otherwise stranded residual oil. This process may also offer the opportunity to recover energy from such oils as methane in future unconventional energy recovery strategies (Grishchenkova et al., 2000; Larter et al., 2005; Gieg et al., 2008, 2010; Jones et al., 2008; Mbadinga et al., 2011).

The composition of the crude oil itself may play a critical role in dictating the extent of degradation of oil. For example, in aerobic seawater microcosms the period before the onset of biodegradation for a fresh crude oil was longer than that observed for the same oil that had been pre-weathered (Atlas and Bartha, 1972). Furthermore, when low molecular weight components were prevented from volatilizing, no biodegradation was observed, suggesting an inhibitory effect of low molecular weight compounds on aerobic oil-degrading microbial consortia (Atlas and Bartha, 1972). Critically, the influence of oil composition on anaerobic oil degradation processes in oil-impacted subsurface systems is currently unknown.

In the present study, anaerobic oil degrading microcosms were either amended with a North Sea crude oil that was artificially weathered to remove volatile aliphatic and aromatic hydrocarbons or amended with the non-weathered oil containing the volatile hydrocarbons. This was done to investigate the inhibitory effects of low molecular weight volatile components of crude oil on the rate and extent of methanogenic oil biodegradation.

## Methods

### Preparation of methanogenic oil-degrading microcosms

Methanogenic microcosms were prepared in glass Wheaton serum bottles (120 ml, VWR Ltd.) and comprised sediment slurry (7 g sediment made up to 100 ml) leaving a headspace of 20 ml. For each microcosm, sediments slurries were prepared with sulfate free carbonate-buffered brackish (7 g/l NaCl) medium (Widdel and Bak, 1992) under anoxic conditions using River Tyne sediment (7 g) as previously described (Jones et al., 2008; Sherry et al., 2009). Subsequently, microcosms were each amended with 250 mg of oil, crimp-sealed and shaken by hand to disperse the oil. Addition of oil was either North Sea crude oil (non-weathered) or North Sea crude oil that was artificially weathered. Weathering was done by incubating non-weathered oil in a glass Petri dish in the air stream of a fume hood at room temperature (0.4 m/s; ~21°C) for 72 h to remove volatile hydrocarbons. Microcosms without oil were prepared to determine the extent of methanogenesis in the absence of crude oil. Control microcosms containing weathered and non-weathered oil were also prepared. The control treatments comprised: 1. addition of bromoethanesulphonic acid (BES, 10 mM final concentration), an inhibitor of methanogenesis and; 2. microcosms which were subjected to three cycles of autoclaving at 121°C, 20 min followed by incubation at 37°C, for ~17 h. This triple autoclaving approach was adopted to promote germination of bacterial spores present in the sediment. Vegetative cells arising from this treatment were then killed by subsequent autoclaving. All the experimental treatments were prepared in biological triplicate and were incubated statically at room temperature (ca. 21°C), in the dark for a total of 1058 days.

### Methane production in microcosms

Methane in the headspace of microcosms was sampled (0.1 ml) using a nitrogen-flushed, gas-tight, push lock syringe (SGE, Australia). Methane concentrations in samples and standards were determined by gas chromatography with flame ionization detection (GC-FID) using a Carlo Erba 5160 GC fitted with a Chrompak Pora plot Q coated fused silica capillary column (30 m × 0.32 mm) with hydrogen as a carrier gas. The oven (35°C) and injection port (250°C) temperatures were fixed. Methane concentrations were determined periodically with reference to external standard calibrations with a standard gas (Scientific & Technical Gases Ltd, Newcastle—under—Lyme, UK). After 345 days, prior to headspace gas analysis, a measured volume of gas were removed from the microcosms using a sterile needle and syringe, to release excess pressure produced by gas production during crude oil biodegradation. The volume of gas removed and the concentration of methane was accounted for when calculating the total mass of methane produced in the microcosms.

### Quantification of volatile hydrocarbons in microcosm headspace

After incubation for 1058 days all microcosms were sacrificed for microbiological and oil chemistry analysis. Prior to sacrificial sampling, volatile hydrocarbons were quantified in headspaces with reference to freshly prepared microcosms comprising North Sea crude oil (250 mg non-weathered oil), distiled water, River Tyne sediment and added deuterated standards (*n*C_5_D_12_–*n*C_10_D_22_ alkanes, methylcyclohexane-D-14, benzene-D-6, toluene-D8, and *o*-xylene-D-10; Cambridge Isotope Laboratories Inc). All microcosms were equilibrated at 20°C in a heated water bath before headspace sampling. Gas chromatography-mass spectrometry (GC-MS) analysis of the headspace gases/vapors was performed on an Agilent 7890A GC split/split less injector (280°C) linked to an Agilent 5975C MSD. Data acquisition was controlled using Chemstation software, in full scan or in selected ion mode for greater sensitivity. The headspace gas sample (100 μl) was injected manually using an SGE gas tight valve syringe. Separation was performed on an Agilent fused silica capillary column (60 m × 0.25 mm i.d.) HP1-MS phase. The GC temperature programme was 30°C for 5 min, ramped to 80°C at 5°C/min and then to 320°C at 25°C/min. The final temperature was held for 5 min. The carrier gas was helium (flow rate of 1 ml/min, initial pressure of 50 kPa, open split at 50 ml/min). Peaks were identified after comparison of their mass spectra with those from the NIST05 library and from their relative retention times.

### Extraction and analysis of oil

Aliquots of sediment slurry (90 ml) were used for oil analysis. Quantification standards (squalane and 1,1′-binaphthyl) were added to the sediment slurry which was saponified (refluxed for 1 h with 1 M KOH in methanol) to allow recovery and analysis of the acid metabolite fraction if required for further analysis. The saponified slurry was acidified to pH ~1 using concentrated HCl. The sediment was then removed by Buchner filtration and liquid-liquid solvent extraction with dichloromethane (DCM) was used to obtain the organic solvent soluble fractions. The hydrocarbon fractions from the sediment slurries and from samples of non-weathered and weathered oils were analyzed by GC. Analytical reproducibility for replicate analyses (*n* = 3) of the C_12_–C_34_ *n*-alkanes in the oil was <1% relative standard error. To quantify *n*-alkanes, aliquots of the organic extracts were separated using a solid phase extraction (SPE) method to provide a total hydrocarbon fraction (Bennett et al., 1996). Alkanes (*n*C_12_–*n*C_34_) and aromatics were quantified from GC analysis of the total hydrocarbon fractions by measuring peak areas relative to the peak area of the added standards.

### Theoretical methane yield from alkane degradation

Theoretical methane yields from the alkanes degraded in the microcosms were derived from the quantity of individual resolved alkanes which were degraded. This was determined by GC analysis of headspace volatile hydrocarbons and solvent extracts from microcosms incubated for 1058 days compared to the oil added at the start of the experiment. The theoretical methane yield was calculated using stoichiometric equations for the methanogenic degradation of each of the alkanes individually. These values were summed to give the total theoretical methane yield from the degraded alkanes (for detailed calculation see Supplementary Methods S1).

### Quantification of *smithella* and *methanomicrobiales* 16S rRNA genes in methanogenic oil-degrading microcosms

DNA extraction from sediment slurry samples (1 ml) was done using a FastDNA Spin Kit for Soil (Q-BIOgene, California, USA) according to the manufacturer's instructions. qPCR assays were performed as previously described using specific primer sets to quantify 16S rRNA gene abundance of total bacteria, *Smithella spp*, hydrogenotrophic methanogens from the order *Methanomicrobiales*, facultative acetoclastic methanogens from family *Methanosarcinaceae*, and obligate acetoclastic methanogens from family *Methanosaetaceae* (Gray et al., 2011; Callbeck et al., 2013). All qPCR assays were performed using a CFX96 real-time PCR detection system (Bio-Rad Laboratories Ltd, Hertfordshire, UK). Typically, DNA template (3 μ l) and appropriate primers (1 μl at a concentration of 10 pmol μl^−1^) were combined with SsoFast EvaGreen Supermix (Bio-Rad). Reaction conditions included an initial denaturation step at 98°C for 3 min, and 40 subsequent reaction cycles which included denaturation (98°C for 5 s), annealing and elongation (58.5°C for 5 s). Standards containing known amounts of target 16S rRNA genes were prepared by serial 10-fold dilution of PCR amplified 16S rRNA genes which were prepared from cloned genes of known sequence, using vector-specific primers (Gray et al., 2011). DNA template-free negative controls were always conducted. PCR efficiencies for all assays were between 70 and 110% and *R*^2^ values for calibration curves were >0.99. Log gene-abundance values for samples all fell within the linear range of the standard calibration.

### Statistical analysis

Biological and chemical data from experimental microcosms were analyzed by One Way analysis of variance (ANOVA) followed by pairwise comparisons of treatments (Tukey HSD), IBM SPSS statistics 19.

## Results

### Methane production in methanogenic oil degrading microcosms

**Figure 1:**
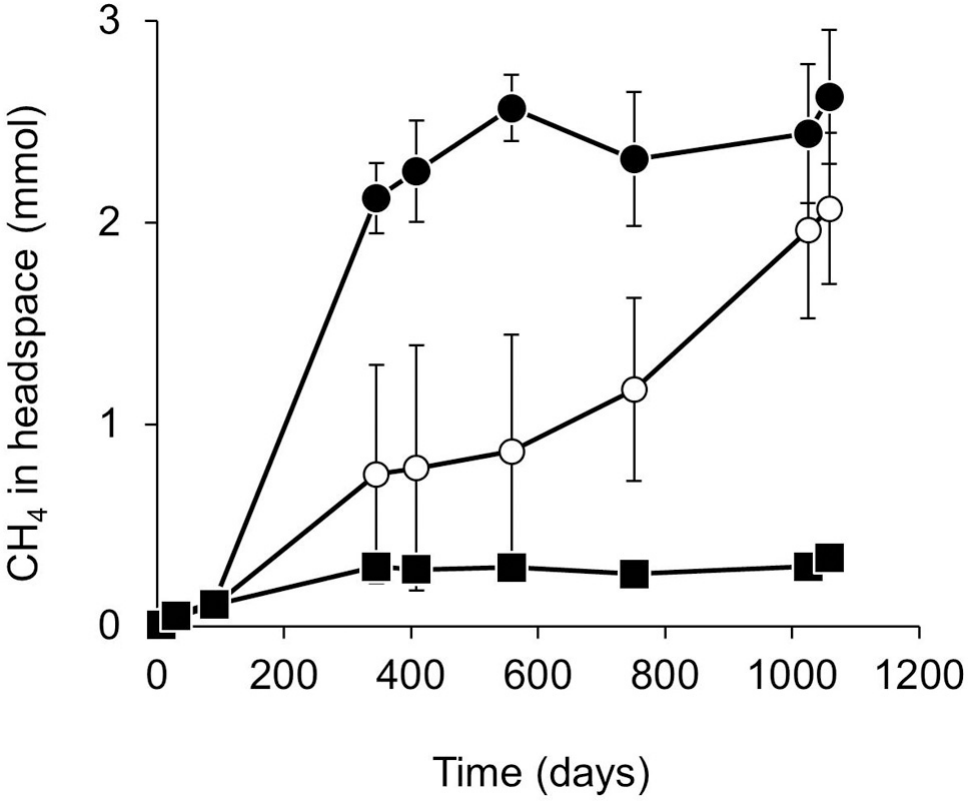
**Methane production from anaerobic microcosms amended with weathered oil (•), non-weathered oil (◦) or prepared without oil (■).** Error bars indicate ±1 × standard error (*n* = 3 oil amended; *n* = 6 without oil).

Methane production was monitored periodically in microcosms amended with weathered oil and non-weathered oil in comparison with unamended controls, as a proxy for methanogenic biodegradation of the crude oil (Figure 1). For the first 90 days, cumulative methane production was not significantly different in the oil-amended and unamended microcosms (*p* > 0.21). By day 345, all oil amended microcosms had higher methane levels than no oil control microcosms (Figure 1). However, from 345 to 751 days, the cumulative methane produced in weathered oil-amended microcosms was significantly higher than in microcosms amended with non-weathered oil (*p* < 0.02). By 1058 days the total amount of methane generated in microcosms with weathered and non-weathered oil was similar (*p* = 0.29). In weathered oil microcosms most methane production had occurred by day 345 after which there was little further accumulation of methane which reached a maximum of 2.7 ± 0.3 mmol (Figure 1; *p* > 0.81). Between 90 and 345 days, the rate of methane production was 1.1 ± 0.1 μmol CH_4_/g wet sediment/day. By contrast methane accumulation in the non-weathered oil microcosms occurred at a much lower rate, reaching a maximum of 2.1 ± 0.4 mmol CH_4_ after 1058 days of incubation (Figure 1). In this case the rate of methane production between 90 and 345 days was 0.4 ± 0.3 μmol CH_4_/g wet sediment/day. The total amount of methane produced in the no-oil control microcosms reached a maximum of 0.29 ± 0.01 mmol by 345 days equating to a rate of 0.12 ± 0.01 μmol CH_4_/g wet sediment/day. In BES-inhibited microcosms the maximum amount of methane produced was 0.06 ± 0.003 mmol CH_4_ in microcosms treated with non-weathered oil and prior to 751 days was 0.05 ± 0.01 mmol CH_4_ in microcosms treated with weathered oil. After 751 days CH_4_ began to accumulate in two of the BES inhibited weathered oil replicates reaching an average of 0.78 ± 0.09 mmol CH_4_ (Supplementary Figure S2). In heat-killed microcosms methane production was consistently very low (0.01 ± 0.001 mmol CH_4_ with non-weathered oil and 0.01± 0.0002 mmol CH_4_ with weathered oil).

### Volatile hydrocarbons in methanogenic oil degrading microcosms

**Figure 2:**
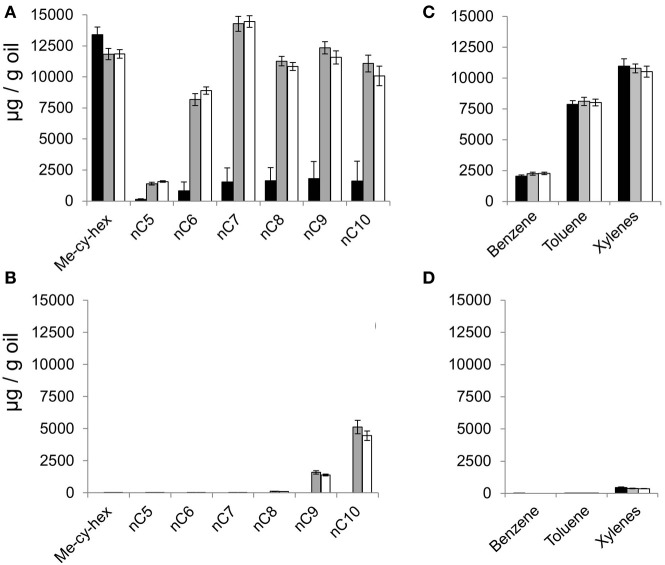
**Headspace volatile hydrocarbons in methanogenic oil-degrading microcosms after 1058 days of incubation.** Headspace saturated hydrocarbons in **(A)** non-weathered oil and **(B)** weathered oil treated microcosms. Headspace aromatic hydrocarbons in **(C)** non-weathered oil and **(D)** weathered oil treated microcosms. Data from oil-amended microcosms are represented by black bars, data from oil-amended and BES-inhibited microcosms are shown as gray bars and data from autoclaved, oil-amended microcosms are shown as white bars. Error bars indicate ±1 × standard error (*n* = 3). Me-cy-hex = methylcyclohexane.

The weathering process significantly (*p* < 0.001) depleted the volatile saturated hydrocarbons compared to the non-weathered oil (*n*C_5_–C_10_, Supplementary Figures S1A,B). By 1058 days, volatile saturated hydrocarbons were present at much higher levels in heat-killed and BES-treated microcosms containing non-weathered oil, compared to methanogenic oil-degrading microcosms that were not heat killed or inhibited (Figure 2A, gray and white bars). In the methanogenic oil degrading microcosms containing non-weathered oil, incubated for 1058 days, the volatile saturated hydrocarbons, with the exception of methylcyclohexane, were present at low concentrations (<1.9 mg/g oil) (Figure 2A, black bars; *p* < 0.001). This indicated that the *n*C_5_–*n*C_10_ alkanes had been microbially degraded during the incubation period. In microcosms treated with weathered oil and incubated for 1058 days, headspace volatile *n*-alkanes were largely absent in the BES-inhibited and heat-killed microcosms (Figure 2B) consistent with the composition of this oil at the start of the experiment (Supplementary Figure S1). Nevertheless all the volatile *n*-alkanes were completely absent in the uninhibited microcosms by 1058 days suggesting that the residual light alkanes i.e., *n*C_9_ and *n*C_10_, not removed by the weathering treatment were most likely degraded to methane (Figure 2B).

By contrast with the *n*-alkanes, the volatile aromatic hydrocarbons (benzene, toluene and xylenes) were present at similar levels in the uninhibited microcosms and in inhibited and heat-killed control microcosms (benzene and toluene *p* ≤ 1.00; xylenes *p* ≤ 0.99; Figure 2C) indicating that none of these aromatic hydrocarbons were degraded under methanogenic conditions. Volatile aromatic hydrocarbons were not present at the end of the incubation period (1058 days) in microcosms treated with weathered oil (Figure 2D) consistent with their absence from the weathered oil (Supplementary Figure S1).

### Biodegradation of moderately volatile alkanes (nC_8_–nC_11_) to methane

**Figure 3:**
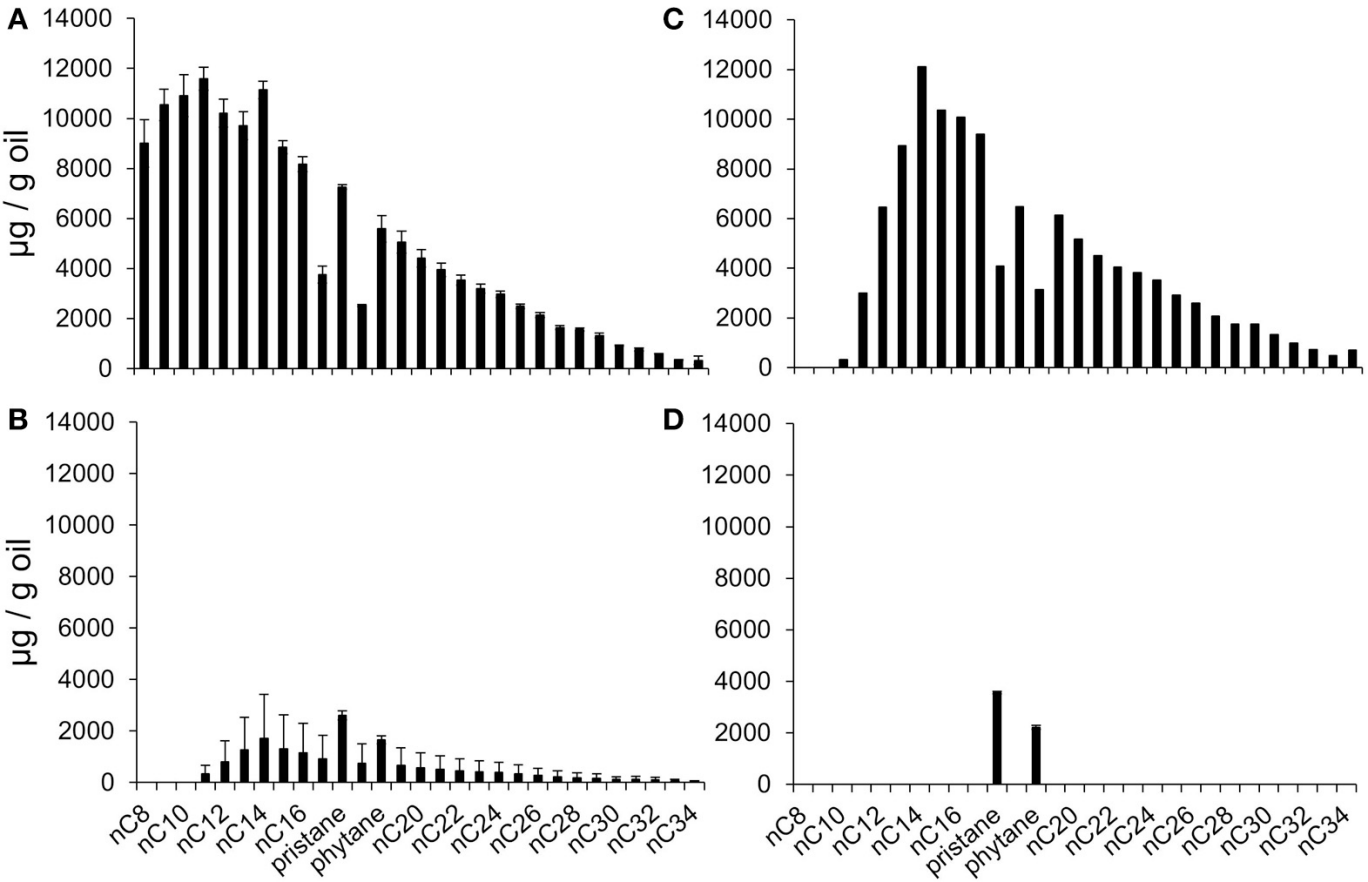
**Saturated hydrocarbons in methanogenic oil-degrading microcosms. (A)** Alkanes in extracts from non-weathered oil treated microcosms at 0 days. **(B)** Alkanes in extracts from non-weathered oil treated microcosms after 1058 days of incubation. **(C)** Alkanes in extracts from weathered oil treated microcosms at 0 days. **(D)** Alkanes in extracts from weathered oil treated microcosms after 1058 days of incubation.

The moderately-volatile alkanes (*n*C_8_–*n*C_11_; vapor pressure 1.5–0.05 kPa at 20°C) present in the non-weathered oil and weathered oil treated microcosms were quantified at the beginning and end of the incubation period (0 days and 1058 days; Figure 3). At the end of the incubation period, most *n*-alkanes in the non-weathered oil microcosms were severely depleted, with *n*C_8_–*n*C_11_ alkanes completely absent (Figure 3B). Given their initial high abundance relative to many of the higher molecular weight alkanes this suggests preferential removal of the lighter *n*-alkanes. *n*-alkanes were completely removed in the weathered oil treated microcosms (Figure 3D) leaving only the branched chain alkanes pristane and phytane, which are more resistant to biodegradation. The ratio of *n*C_17_:pristane in oil treated microcosms relative to autoclaved controls after 1058 days incubation and oils added at the start of the experiment (Day 0), also indicated that biodegradation of *n*-alkanes had occurred in oil-treated microcosms (*n*C_17_:pristane–Non-weathered oil Day 0, 1.95 ± 0.15; heat-killed 1058 days, 1.98 ± 0.05; live microcosms 1058 days, 0.32 ± 0.32; Weathered oil heat-killed 1058 days, 1.75 ± 0.06; live microcosms 1058 days, <0.01 ± 0.00). The *n*C_17_:pristane ratio in the heat-killed control amended with weathered oil was slightly reduced during the incubation period (1.75 ± 0.06 compared to 1.95 ± 0.15, *p* = 0.03) suggesting that the heat-treatment may not have been completely effective in inactivating all of the *n*-alkane degrading bacteria present in the sediment.

### Stoichiometry of methane production from alkane degradation

The theoretical methane yields derived from the observed degradation of the *n*-alkanes in the weathered oil was 1.6 mmoles. This is somewhat less than the average measured methane (2.4 ± 0.3 mmoles) which was attributable to oil degradation i.e., after subtraction of the methane yield of the no oil controls. However, the *n*-alkanes resolved and quantified by GC and included in this yield estimate, do not constitute the entire complement of degradable alkanes present in the oil (Larter et al., 2012). The theoretical methane yield from *n*-alkanes degraded in the non-weathered oil was 2.2 mmoles which did match with measured maximum methane concentrations in these microcosms (2.1 ± 0.4 mmol). By contrast, aromatic hydrocarbons such as methylnaphthalenes and methylbiphenyls were not significantly biodegraded in the experimental microcosms relative to the controls. Typically 4-methylbiphenyl (4-MB) is degraded preferentially to 3-methylbiphenyl (3-MB) and 2-methylnaphthalene (2-MN) is degraded preferentially to 1-methylnaphthalene (1-MN), but the 4-MB/3-MB and 2-MN/1-MN ratios changed little over the period of incubation (Supplementary Table S1).

### qPCR analysis of 16S rRNA genes (*smithella* and *methanomicrobiales*)

**Figure 4:**
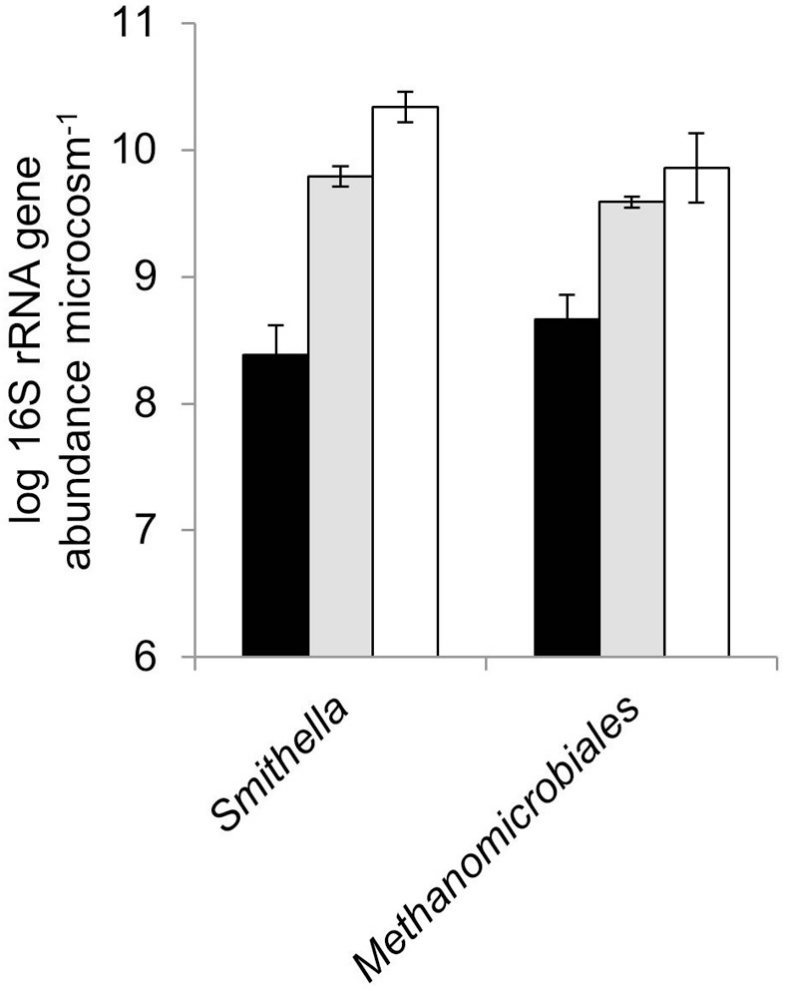
**Log abundance of 16S rRNA genes from *Smithella* spp. and methanogens from the order *Methanomicrobiales* in microcosms amended with weathered oil (gray bars), non-weathered oil (white bars) or prepared without oil (black bars).** All data are from microcosms incubated for 1058 days. The abundance of 16S rRNA genes from *Smithella* spp. and methanogens from the order *Methanomicrobiales* at the start of the incubation was 7.00 ± 0.1 and 6.91 ± 0.24 log 16S rRNA genes/microcosm respectively. Error bars indicate ±1 × standard error (*n* = 3).

*Smithella* spp. and methanogens from the order *Methanomicrobiales* are known to be important in methanogenic crude oil biodegradation in systems similar to those studied here (Jones et al., 2008; Gray et al., 2011). The abundance of 16S rRNA genes from these groups of organisms was therefore quantified in the methanogenic oil degrading microcosms following 1058 days of incubation (Figure 4). Bacteria from the genus *Smithella*, implicated in syntrophic alkane degradation were significantly enriched in both the weathered oil and non-weathered oil amended microcosms relative to no oil controls (*p* < 0.005). 16S rRNA genes from *Smithella* spp. were enriched by 1.4 ± 0.3 and 2.0 ± 0.3 log units in weathered and non-weathered oil containing microcosms respectively, compared with control microcosms with no added oil. Nevertheless, there was not a significant difference between the abundance of 16S rRNA genes in microcosms amended with weathered and non-weathered oil (*p* = 0.197). This is consistent with the observation that similar amounts of methane were produced in both the non-weathered and weathered oil microcosms (*p* = 0.29). In addition, 16S rRNA genes from hydrogenotrophic methanogens of the order *Methanomicrobiales* were significantly enriched in the non-weathered oil and weathered oil amended systems relative to unamended controls (1.2 ± 0.2 and 0.9 ± 0.2 log units respectively; *p* < 0.006). Again there was not a significant difference between the weathered and non-weathered oil microcosms (*p* = 0.373), consistent with the similar amount of methane produced in both the non-weathered and weathered oil microcosms (*p* = 0.29). Total bacteria, facultative acetoclastic methanogens (Family *Methanosarcinaceae*) and obligate acetoclastic methanogens (Family *Methanosaetaceae*) were also quantified by qPCR. No significant differences were observed between pairwise comparisons of 16S rRNA gene abundances from DNA extracts from microcosms prepared with weathered oil, non-weathered oil or prepared without oil for total bacteria (*p* ≥ 0.548), family *Methanosarcinaceae* (*p* ≥ 0.697) or family *Methanosaetaceae* (*p* ≥ 0.051) (Supplementary Figure S3).

## Discussion

The inhibitory effect of volatile compounds in crude oil on aerobic hydrocarbon degradation has been known for some time (Atlas and Bartha, 1972; Atlas, 1975). More recent studies suggest that low molecular weight *n*-alkanes, alicyclic alkanes and monoaromatic hydrocarbons may also inhibit anaerobic processes (Chen et al., 2008). Fermentative growth of *Acetobacterium woodii* was completely inhibited by short chain alkanes (<C_9_), whereas, with increasing chain length, above C_9_, inhibition decreased (Rodriguez Martinez et al., 2008). Toluene has also been shown to inhibit acetoclastic methanogenic activity in anaerobic reactor sludge (Ince et al., 2009) and, BTEX compounds are known to inhibit the anaerobic biodegradation of each other (Dou et al., 2008). Their inhibitory properties are thought to be due to their water solubility which renders them both bioavailable for degradation but at the same time more toxic because the accumulation of such compounds causes the cell membrane to swell and leak causing lysis (Chen et al., 2008; Rodriguez Martinez et al., 2008).

Many laboratory studies have documented methane production from the degradation of crude oil and individual or mixtures of hydrocarbons (Muller, 1957; Zengler et al., 1999; Townsend et al., 2003; Siddique et al., 2006, 2007; Gieg et al., 2008, 2010; Jones et al., 2008; Wang et al., 2011; Zhou et al., 2012). Methane production profiles from many of these studies share common characteristics including an initial lag phase varying between 3 and 6 months followed by extensive hydrocarbon-driven methane production on time scales of 100's of days. It should be noted that in the first of these studies in the 1950s (Muller, 1957) it was suggested that only “higher alkanes” above C_16_ were degradable. In fact a very broad range of *n*-alkanes are now known to be utilized by methanogenic consortia (Townsend et al., 2003; Siddique et al., 2006; Jones et al., 2008; Zhou et al., 2012) including those below C_16_, and all these *n*-alkanes are removed at faster rates than aromatic oil components (Townsend et al., 2003; Jones et al., 2008). In the current study *n*C_5_–*n*C_34_ alkanes were removed without alteration of volatile aromatic hydrocarbons and methylcyclohexane within the time frame of the experiment (Figures 2, 3). Moreover there was no evidence for the removal of higher molecular weight aromatic hydrocarbons such as methylated naphthalenes and methylbiphenyls (Supplementary Table S1). These findings are consistent with patterns observed in subsurface environments such as oil reservoirs where alkane degradation is typically considered to precede the degradation of aromatic compounds (Larter et al., 2012). In in-reservoir degraded oils, removal of *n*C_6–15_ *n*-alkanes before C_15+_ *n*-alkanes is observed (Larter et al., 2012). This is supported by our study of the degradation of the non-weathered oil where the most abundant residual *n*-alkanes were *n*C_11_+, despite higher initial abundance of lighter alkanes (Figure 3B). For these lighter alkanes though, the size defined order of degradation is likely reversed (*n*C_10_ > *n*C_8_ > *n*C_7_ > *n*C_6_) (Siddique et al., 2006) an observation either attributable to the water solubility/hydrophobicity of the compounds whose octanol/water partition coefficients (P_ow_) decrease with decreasing chain length, or due to selective uptake of the more hydrophobic alkanes across cell membranes of *n*-alkane-degrading microorganisms (Siddique et al., 2006).

The persistence of benzene, methylcyclohexane, toluene, and xylene (vapor pressure range from 9.9 to 0.8 kPa at 20°C) but loss of the *n*C_7_–*n*C_10_ alkanes (vapor pressure range from 5.3 to 0.1 kPa at 20°C) in the methanogenic non-weathered oil treated microcosms further demonstrates that volatile hydrocarbon removal was biological and not due to evaporation during sampling of headspace. A direct comparison of heptane and methylcyclohexane, for instance, indicates that while they have almost identical vapor pressures (5.3 and 4.9 kPa at 20°C respectively) methylcyclohexane persists while heptane was removed in triplicate microcosms subjected to the same headspace sampling regimen (Figure 2A).

It has been previously suggested that the composition of an oil controls its susceptibility to methanogenic degradation (Muller, 1957) and, specifically, based on a comparison of the degradation of a weathered and unaltered “light paraffinous oil” it was inferred that volatile aromatics are likely inhibitory. In the present study we have now quantified the initial abundances and fates of individual compounds in such oils and clearly shown that methanogenic removal of alkanes proceeded more rapidly when volatile hydrocarbons are absent. Based on our data, light *n*-alkanes, methyl-cyclohexane or aromatic hydrocarbons present in the non-weathered oil but absent in the weathered oil, can all be implicated in the inhibition, although, it is clear that the light *n*-alkanes were utilized by the methanogenic consortia in our experiments while the light aromatic hydrocarbons were not. This contrasts with the observation that BTEX are effectively degraded under methanogenic conditions in mature fine tailings from oil sands processing facilities where naphtha containing high levels of low molecular weight alkanes and aromatic hydrocarbons are used in upgrading of heavy oil (Siddique et al., 2007). Degradation of aromatics (^13^C-labeled 2-methylnapthalene) at a rate greater than that for degradation of *n*-alkanes (^13^C-labeled hexadecane) was reported in enrichments prepared with production waters from the Dagang oil field (Jiménez et al., 2012). Interestingly, the study reports that most of the oil from Dagang is heavy oil which was almost completely depleted of *n*-alkanes (92–100% alkane degradation in 8 Dagang oils), therefore the microorganisms in production waters from the Dagang oil field which were used as inoculum in microcosm experiments may already have been pre-enriched for aromatic hydrocarbon degraders in the reservoir and therefore show a more rapid degradation of aromatic hydrocarbons (2-methylnaphthalene) than alkanes (n-hexadecane). Furthermore, some of the enrichments were reported to have received additions of 2 mM sulfate (Jiménez et al., 2012), and it has been shown previously that under sulfate-reducing conditions the extent of aromatic hydrocarbon degradation (3-methylbiphenyl) is greater when sulfate is present, than under methanogenic conditions (Jones et al., 2008).

*Smithella* and *Methanomicrobiales* were significantly enriched in both the weathered oil and non-weathered oil amended microcosms relative to controls with no oil addition after 1058 days (Figure 4), supporting previous research that they play an important role in alkane biodegradation (Jones et al., 2008; Gray et al., 2011). No differences were observed between the abundance of *Smithella* and *Methanomicrobiales* in weathered oil amended microcosms compared to non-weathered oil amended microcosms suggesting that weathering of the oil does not adversely affect the microbiology of oil biodegradation after prolonged periods of >1000 days. In agreement with data from (Jones et al., 2008), the 16S rRNA gene abundances of facultative (*Methanosarcinaceae*) and obligate acetoclastic methanogens (*Methanosaetaceae)* were lower than hydrogenotrophic methanogens (*Methanomicrobiales)* in the oil-amended microcosms.

### Implications for anaerobic bioremediation and energy recovery technologies

In surface oil spills which are common throughout the world, knowledge of the composition of an oil, its distribution in the environment and, the specific local environmental risks associated with a spill can inform remediation strategies (Atlas and Bragg, 2009; Atlas and Hazen, 2011). Monitored natural attenuation (MNA) is a risk based approach where the likelihood that a sensitive receptor will be impacted by a contaminant spill is assessed on the basis of natural biodegradation rates and other factors such as contaminant transport. Under oxic conditions rates of crude oil degradation in marine sediments can be enhanced by the application of fertilizers and dispersants, strategies that have been used for large oil spills such as the Exxon Valdez and the Deepwater Horizon (Atlas and Hazen, 2011). The release of petroleum components into anaerobic subsurface environments is also common throughout the world arising from natural releases, leaks, or deliberate disposal to soils, marine and freshwater sediments and aquifers. MNA is an accepted option for management of such contaminated environments (Parisi et al., 2009; Feisthauer et al., 2012; Naidu et al., 2012); indeed microbially mediated natural attenuation is likely even more important in the subsurface because processes such as evaporative loss and photooxidation are respectively either limited or entirely absent. It has been shown that methanogenic degradation of crude oil hydrocarbons can be significant in polluted subsurface environments (e.g., the Weißandt-Gölzau and Bemidji aquifers) and further that release of methane from this process leads to the depletion of other electron acceptors as a result of anaerobic and aerobic methane oxidation (Amos et al., 2005; Feisthauer et al., 2010, 2012). Indeed the wide distribution of methanogenic petroleum hydrocarbon degradation capacity in both shallow and deep subsurface environments suggests that this process will be important after the depletion of other electron acceptors, especially if, as has been suggested, methanogenic consortia are more tolerant to higher concentrations of hydrocarbon contaminants compared to microorganisms using other electron acceptors, such as the sulphate-reducing bacteria (Baker et al., 2012).

Despite the documented importance of methanogenic oil degradation in subsurface environments we have shown that hydrocarbon degradation in methanogenic crude oil impacted environments may be impeded by some components of the crude oil. Oils with higher levels of volatile hydrocarbons will be more inhibitory to oil-degrading microbial consortia than weathered oils with little or no volatiles and this inhibition will consequently reduce the rate of contaminant biodegradation. It is therefore important that the presence of volatile hydrocarbons is taken into account when MNA is applied for the management of contaminant plumes in anoxic environments.

In addition to remediation, methanogenic petroleum hydrocarbon degradation offers the potential to enhance energy recovery from stranded oil in petroleum reservoirs as methane (Grishchenkova et al., 2000; Gieg et al., 2008, 2010; Jones et al., 2008). This might be achieved by stimulation of indigenous methanogenic oil-degrading communities or specific methanogenic oil-degrading consortia. Based on the data presented here it appears that the chemical composition of a crude oil or other hydrocarbon mixtures may have marked effects on oil degradation to methane. Thus oils with higher levels of volatile hydrocarbons in environments where no alternative mechanisms of volatile hydrocarbon loss are possible, may be less amenable to rapid anaerobic conversion to methane. Determination of the isotopic composition of methane in the incubations would potentially have been valuable to this study, as used previously in a hydrocarbon-degrading methanogenic enrichment culture (Morris et al., 2012) and in the assessment of *in situ* processes in hydrocarbon contaminated aquifers (Feisthauer et al., 2010, 2012). Future work should quantitatively determine the absolute and relative inhibitory effects of individual light hydrocarbons or defined mixtures to allow accurate prediction of timescales of subsurface methanogenic oil biodegradation under natural and engineered conditions.

### Implications for the dynamics of petroleum reservoir degradation and heavy oil formation

In petroleum reservoirs, oil composition is controlled by a complex interplay of geological factors i.e., source rock variation, thermal maturity, oil expulsion and migration and the balance between oil degradation and fresh oil charge (Larter et al., 2005, 2012; Adams et al., 2006). This complexity gives rise to oils which have different absolute amounts and relative distributions of saturated and aromatic hydrocarbons, resins and asphaltenes. For deep subsurface petroleum accumulations putatively inhibitory volatile compounds can often only be removed by water washing or biodegradation and thus conditions conducive to petroleum biodegradation and water washing (reservoir temperatures less than 80–90°C; presence of nutrients and an extensive oil-water contact zone Head et al., 2003) may enhance the removal of potentially inhibitory compounds and thus accelerate biodegradation. In the context of water washing it is also worth noting that the volatile hydrocarbons have lower octanol water partition coefficients than higher molecular weight hydrocarbons.

From the current study, it can be inferred that the onset and subsequent rates of methanogenic hydrocarbon degradation will be dependent in part on the initial concentration of volatile hydrocarbons in reservoired oil, as well as on the availability of nutrients, water, and transport/diffusion rates. Oils with high concentrations of light *n*-alkanes and aromatic hydrocarbons would retard degradation of all other compound classes. In this study it seems that much of the volatile hydrocarbon component is eventually degraded in the non-weathered oil (Figure 2A) and this is likely to occur much more slowly under reservoir conditions. Indeed *in situ* rates of oil degradation inferred from reservoir field data indicate that in reservoir degradation rates are substantially lower (first order rate constants of 10^−6^–10^−7^ year^−1^) than the rates measured in laboratory incubations (Larter et al., 2003). Thus if all rates are scaled to in-reservoir degradation timescales, then a difference in lag phase between a weathered and a non-weathered oil may well be significant. Furthermore, in-reservoir oil degradation typically occurs on similar time-scales to oil charging and mixing (Larter et al., 2012) thus inhibitory compounds may be constantly replenished during continuous charging and mixing resulting in a subtle control on the rate of in-reservoir oil biodegradation.

The discovery of the inhibitory effects of volatile hydrocarbons on methanogenic crude oil biodegradation is also relevant to the “biostatic hypothesis” (Sunde and Torsvik, 2005). The biostatic hypothesis is based on the observation that, despite being replete with appropriate electron donors, electron acceptors and nutrients, samples from petroleum reservoirs do not exhibit souring attributable to the activity of sulfate-reducing bacteria unless they have been degassed or diluted (Sunde and Torsvik, 2005). It is possible that the inhibition of microbial activity by volatile hydrocarbons that we observed in this study may at least, in part, explain the apparent biostatic nature of some petroleum reservoirs.

To our knowledge this is the first systematic evaluation of the inhibition of methanogenic biodegradation of crude oil by volatile hydrocarbons. The geochemical and microbiological findings have implications for understanding rates of in-reservoir oil biodegradation and the formation of heavy oil. The presence of volatile hydrocarbons should also be considered when monitored natural attenuation or biostimulation is applied for the management of hydrocarbon plumes in contaminated anoxic environments. Furthermore, knowledge of the chemical composition of a crude oil may serve to enhance energy recovery from stranded oil as methane, in petroleum reservoirs.

## Author contributions

Ian M. Head, D. Martin Jones, and Neil D. Gray developed the concept and designed experiments; Angela Sherry prepared and analyzed microcosm experiments. Angela Sherry and Russell J. Grant conducted molecular microbiology and analyzed data with Neil D. Gray; Data interpretation was conducted by Neil D. Gray, Angela Sherry, and Ian M. Head; Carolyn M. Aitken performed oil geochemistry and analyzed the data together with D. Martin Jones; Angela Sherry, Neil D. Gray, and Ian M. Head wrote the manuscript.

## Conflict of interest statement

The authors declare that the research was conducted in the absence of any commercial or financial relationships that could be construed as a potential conflict of interest.
