# Supplementary material for: Volatile hydrocarbons inhibit methanogenic crude oil degradation
Source: Front Microbiol. 2014 Apr 3;5:131. doi: 10.3389/fmicb.2014.00131 (PMC3982060; doi:10.3389/fmicb.2014.00131)
Supplement: Supplementary file 1 [file DataSheet1.DOCX]

Volatile hydrocarbons inhibit methanogenic crude oil degradation.

Angela Sherry, Russell J. Grant,^†^ Carolyn M. Aitken, D. Martin Jones, Ian M. Head^*^ and Neil D. Gray.

School of Civil Engineering & Geosciences, Newcastle University, Newcastle upon Tyne, UK.

**Correspondence:**

Professor. Ian M Head

Newcastle University

School of Civil Engineering & Geosciences

Room 3.16, Devonshire Building

Devonshire Terrace

Newcastle-upon-Tyne, NE1 7RU, UK

[Ian.head@ncl.ac.uk](mailto:Ian.head@ncl.ac.uk)

**Supplementary Information:**

Number of pages 6

Number of Figures 3 – Supplementary Figure S1, Supplementary Figure S2, Supplementary Figure S3

Number of Tables 1 – Supplementary Table S1

Supplementary Methods – Supplementary Methods S1.


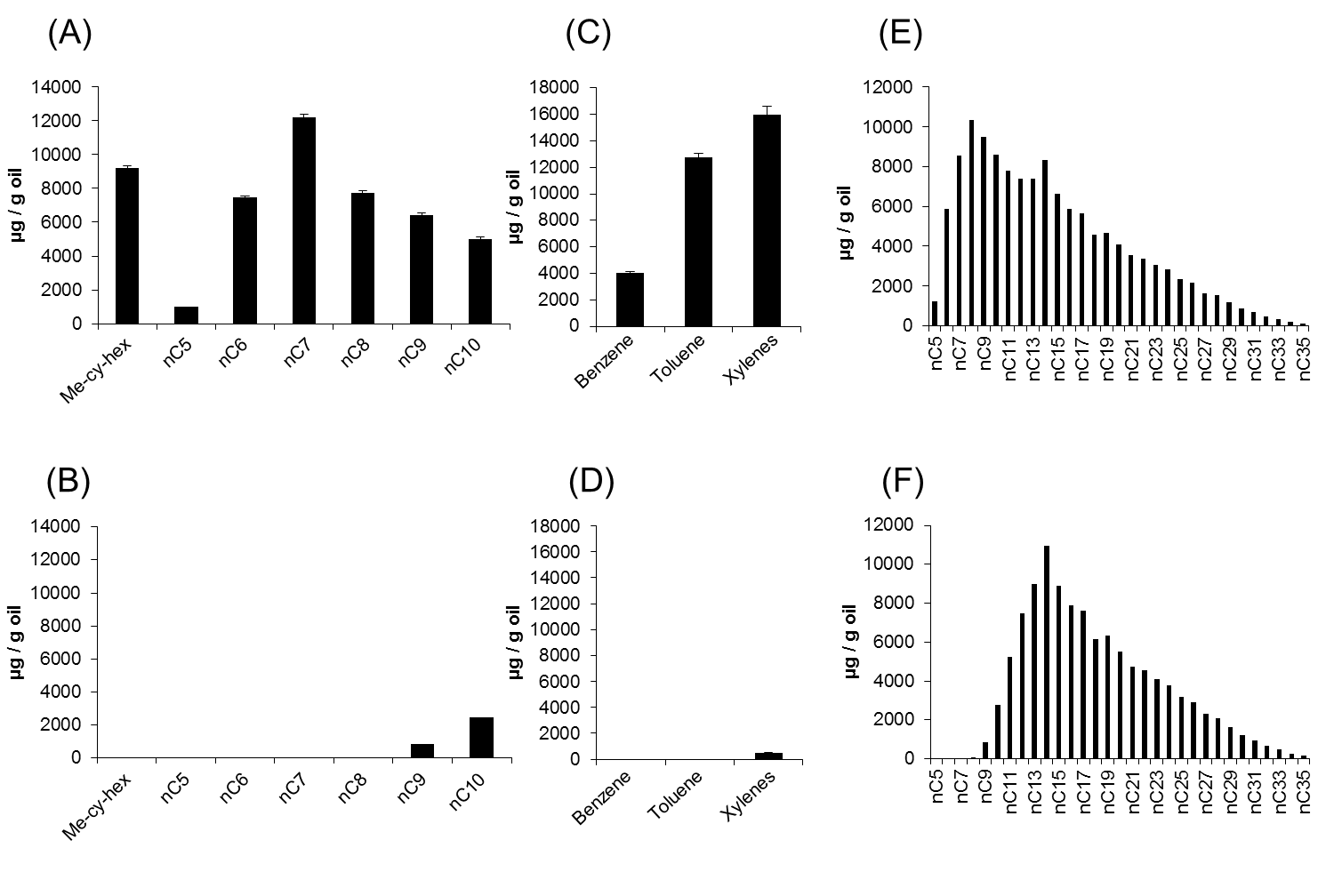


Supplementary Figure S1. Composition of the volatile saturated hydrocarbon fraction ((A) and (B), *n*C_5_-*n*C_10_), the volatile aromatic hydrocarbon fraction ((C) and (D)) and higher molecular weight saturated hydrocarbon fraction ((E) and (F), *n*C_5_-*n*C_35_) in oils used to amend anaerobic microcosms. (A), (C) and (E); an non-weathered North Sea crude oil and (B), (D) and (F); the same North Sea crude oil following artificial weathering.

Supplementary Table S1. Ratio of 4-methylbiphenyl (4-MB) to 3-methylbiphenyl (3-MB) and 2-methylnaphthalene (2-MN) to 1-methylnaphthalene (1-MN) in weathered oil treated microcosms and non-weathered oil treated microcosms relative to autoclaved controls after 1058 days incubation and oils added at the start of the experiment (0 days).

|  | Ratio  4-MB/3-MB (± S.E.) | p-value | Ratio  2-MN/1-MN (± S.E.) | p-value |
| --- | --- | --- | --- | --- |
| Non-weathered oil, 0 days (n=1) | 0.30 |  | 0.78 |  |
| Non-weathered oil, 1058 days (n=3) | 0.32 (± 0.015) | 0.354* | 0.80 (± 0.004) | 0.460* |
| Non-weathered oil killed, 1058 days (n=3) | 0.29 (± 0.004) |  | 0.89 (± 0.054) |  |
|  |  |  |  |  |
| Weathered oil, 0 days (n=1) | 0.30 |  | 0.89 |  |
| Weathered oil, 1058 days (n=3) | 0.34 (± 0.010) | 0.354* | 0.84 (± 0.010) | 0.227* |
| Weathered oil killed, 1058 days (n=3) | 0.31 (± 0.013) |  | 0.88 (± 0.016) |  |

*Significance of one-way ANOVA analysis of ratio in oil-amended microcosms in comparison to heat-killed, oil-amended microcosms

Supplementary Figure S2. Methane production in BES-inhibited microcosms containing artificially weathered oil (closed squares) and non-weathered oil (closed diamonds).

Supplementary Figure S3. Log abundance of 16S rRNA genes from total bacteria and acetoclastic methanogens from the families *Methanosarcinaceae* and *Methansaetaceae* in microcosms amended with weathered oil (grey bars), non-weathered oil (white bars) or prepared without oil (black bars). All data are from microcosms incubated for 1058 days. Error bars indicate ±1 x standard error (n=3).

Supplementary Methods S1. Calculation of theoretical methane yield from alkane degradation

Theoretical methane yields from the alkanes degraded in the microcosms were derived from the quantity of individual resolved alkanes which were degraded. This was determined by GC analysis of headspace volatile hydrocarbons and solvent extracts from microcosms incubated for 1058 days compared to the oil added at the start of the experiment. The theoretical methane yield was calculated using stoichiometric equations for the methanogenic degradation of each of the alkanes individually. These values were summed to give the total theoretical methane yield from the degraded alkanes.

The stoichiometry of conversion of each individual alkane to methane and CO_2_ was determined for each of the C_5_ – C_34_ alkanes, following the generic calculation

8C_x_H_y_ + (8x – 2Y)H_2_O → (4x + Y)CH_4_ +(4X -Y)CO_2_

(According to Dolfing J, Xu A, Gray ND, Larter SR, Head IM. The thermodynamic landscape of methanogenic PAH degradation. Microbial Biotechnology 2009, 2(5), 566-574).

e.g. C_5_H_12_ + 2H_2_O >>>>> 4CH_4_ + 1CO_2_ (80% of the carbon is converted to methane)

4C_16_H_34_ + 30H_2_0 >>>>> 49CH_4_ + 15CO_2_ (76.5% of carbon is converted to methane)

4C_34_H_70_ + 66H_2_O >>>>>> 103 CH_4_ + 33CO_2_ (75.7% of carbon is converted to methane)

In addition for each alkane the proportional carbon composition (by weight) was calculated using the generic formula =12*(Cn)/(12*Cn + (Cn*2) +2)

e.g. C_5_H_12_ = 0.833

C_16_H_34_ = 0.850

C_34_H_70_ = 0.854

Using these values and the quantitative data for each individual alkane (µg/g oil) a theoretical yield of methane was separately calculated for the initial weathered and non-weathered oils and for the residual alkanes left in microcosms at the end of the 1058 day degradation experiment. Calculations were based on an assumption of the complete conversion of the alkanes present in to CH_4_ and CO_2_. For volatile alkanes estimates of the mass of individual alkanes (µg/g oil) were derived from headspace analysis measurements.

Theoretical methane yields were based on the calculations:

Number of mmoles of methane obtained from an individual alkane = ((mass of alkane in mg (in 0.25 g oil) *proportion of C in the alkane)*proportion of C stoichiometrically converted to methane))/12

Number of moles of methane from total alkanes = sum of all the moles of methane obtained from the individual alkanes
